# Antiviral Properties of ISG15

**DOI:** 10.3390/v2102154

**Published:** 2010-09-28

**Authors:** Deborah J. Lenschow

**Affiliations:** 1 Department of Medicine, Washington University School of Medicine, St. Louis, MO 63110, USA; E-Mail: dlenscho@dom.wustl.edu; Tel.: +314-362-8637; Fax: +314-454-1091; 2 Department of Pathology and Immunology, Washington University School of Medicine, St. Louis, MO 63110, USA

**Keywords:** ISG15, interferon, antiviral, ubiquitin-like molecule

## Abstract

The type I interferon system plays a critical role in limiting the spread of viral infection. Viruses induce the production of interferon (IFN), which after binding to the IFN-α/β receptor (IFNAR), and triggering of the JAK/STAT signaling cascade, results in the induction of interferon-stimulated genes (ISGs). These ISGs function to inhibit viral replication and to regulate the host immune response. Among these ISGs, the ubiquitin-like molecule, ISG15, is one of the most strongly induced proteins. Similar to ubiquitin, through an IFN induced conjugation cascade, ISG15 is covalently linked to a variety of cellular proteins, suggesting regulation of different cellular processes. Studies performed over the past several years have shown that ISG15 plays a central role in the host’s antiviral response against many viruses. Mice lacking ISG15 display increased susceptibility to multiple viruses. Furthermore, several viruses have developed immune evasion strategies that directly target the ISG15 pathway. Work is now underway to determine the mechanism by which ISG15 functions as an antiviral molecule, such that therapies targeting this pathway can be developed in the future.

## 1. Introduction—The ISG15 Pathway

ISG15 was first noted in type I interferon (IFN; IFN-α, -β) treated cell lysates in 1984 and was soon after identified as having sequence homology to ubiquitin (reviewed in reference [1]) [2,3]. The crystal structure of ISG15 revealed that it is composed of two domains, each of which assumes a β-grasp fold similar to the ubiquitin structure [2]. ISG15 is one of the most abundantly-induced transcripts upon type I IFN treatment, as well as following TLR ligation and viral infection [3,4]. It is synthesized as a 17 kDa precursor protein that is proteolytically processed into its mature 15 kDa form [3]. Following stimulation with type I IFN, ISG15 exists in three distinct states- free within the cell, released into the extracellular space, or conjugated to target proteins.

Early after its identification, ISG15 was reported to be found in an extracellular form. Stimulation of human monocytes with type I IFN resulted in as much as 50% of the accumulated ISG15 being released into the media [5]. Furthermore, the repeated treatment of patients with interferon-β_ser_ resulted in detectable levels of ISG15 in their sera within 48 hours of treatment [5]. ISG15 doesn’t contain a signal sequence; therefore, the mechanism by which it is released remains unknown. Several immunomodulatory activities have been attributed to recombinant or extracellular ISG15 including its ability to induce NK cell proliferation, augment lymphokine-activated-killer (LAK) activity, stimulate the production of IFN-γ, induce DC maturation, and to function as a chemotactic factor for neutrophils [6–8]. To date no cell surface receptor has been identified that binds to ISG15; therefore the role that its potential cytokine activity plays *in vivo* is yet to be ascertained.

**Figure 1:**
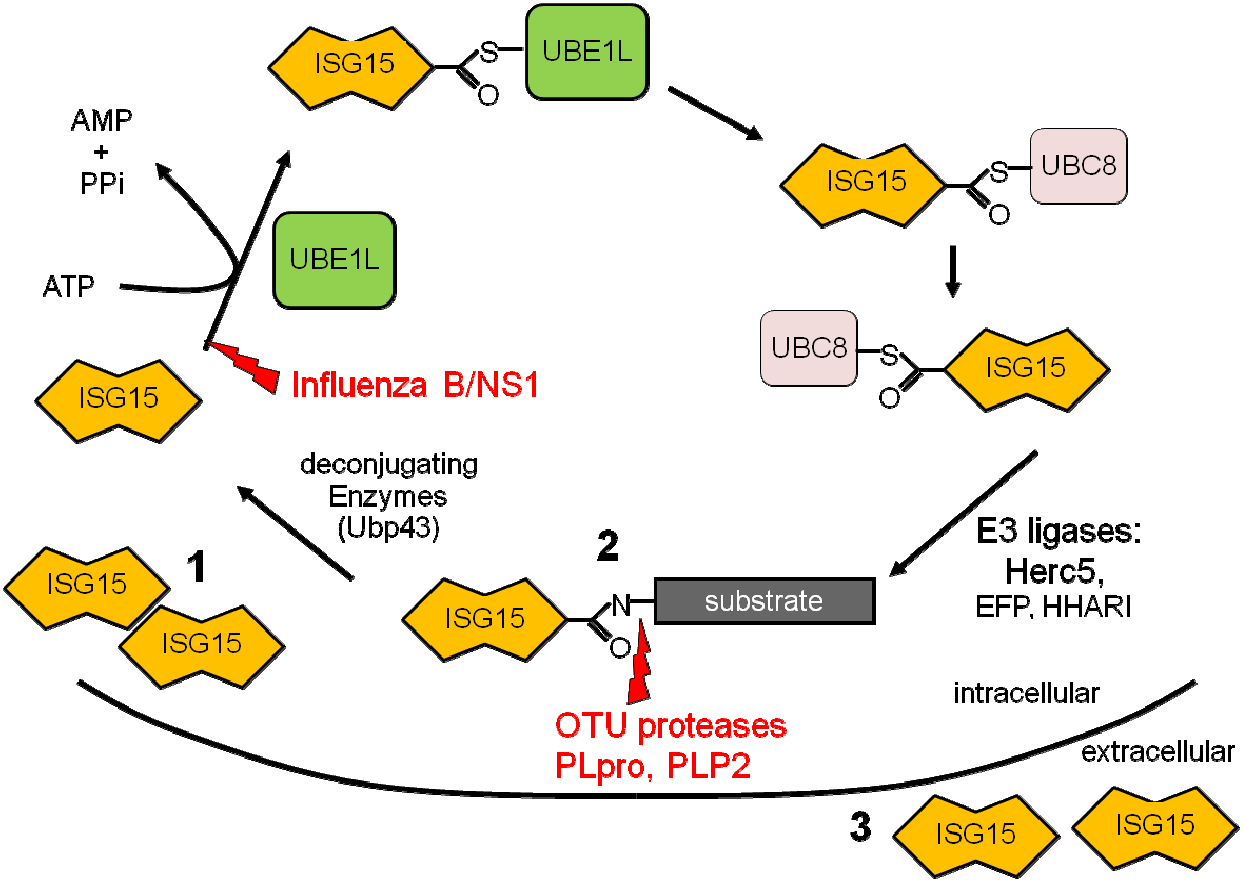
ISG15 conjugation cascade. Following exposure to IFN a cell will upregulate free intracellular ISG15 **(1)**, conjugate ISG15 to target proteins **(2)**, and release ISG15 into the extracellular space **(3)**. Shown in red are the immune evasion strategies utilized by viruses to circumvent the ISG15 pathway.

Similar to ubiquitin, the mature form of ISG15 contains a C-terminal LRLRGG motif that mediates its covalent conjugation to target proteins, termed ISGylation [9,10]. Within two hours after type I IFN treatment, free ISG15 is detectable in cell lysates, followed by conjugate formation 12–16 hours later [9]. ISGylation utilizes a mechanism similar to ubiquitin, requiring three enzymatic steps (Figure 1). The activating E1 enzyme, Ube1L, was initially identified by Robert Krug and colleagues and functions only in the ISG15 pathway, forming an ATP-dependent thioester bond with ISG15 [11]. The recent generation of an UbE1L knockout mouse has confirmed its role as the ISG15 E1. These mice express free ISG15, but do not form ISG15 conjugates following LPS stimulation or during viral infection [12]. Following activation, ISG15 is transferred to the active-site cysteine of the conjugating E2 enzyme UbcM8/UbcH8 [13,14]. This is followed by the transfer of ISG15 to lysine residues on target proteins by the E3 ligases. To date, three E3 ligases have been shown to interact with the ISG15 pathway and include human HERC5 (Ceb1)/mouse HERC6, HHARI, and Efp [15–20]. ISGylation is reversible due to specific removal of ISG15 from conjugated proteins by the deconjugating enzyme Ubp43 (USP18) [21,22]. A screen for additional deubiquitinating enzymes that could target ISG15 conjugates identified USP2, USP5, USP13, and USP14 as potential candidates, whose significance remain to be determined *in vivo* [23]. While in many ways the ISG15 pathway parallels the ubiquitin conjugation pathway, there are differences that exist. First, all members of the ISG15 conjugation cascade are upregulated by type I IFNs. Second, unlike ubiquitin, in which hundreds of E3s are responsible for the individual substrate specificity, in the ISG15 pathway, Herc5 appears to be a dominant E3 ligase that coordinates the conjugation of the majority of substrates to ISG15. The inhibition of Herc5 expression with siRNA abrogated greater than 90% of the ISG15 conjugate formation that was induced [15]. A recent study by Durfee and colleagues has shown the Herc5 associates with polyribosomes and appears to target newly synthesized proteins, including viral proteins, for modification by ISG15, helping to explain the broad substrate specificity of Herc5 [24].

Numerous screens have identified nearly 200 candidate ISG15 targets which span a diverse array of biological processes, including protein translation, cell cycle regulation, signal transduction, glycolysis, cell motility, and immune regulation [25–28]. These targets suggest that ISG15 may be involved in the regulation of a variety of host processes. This has been recently reviewed elsewhere [1]. However, it is still largely unknown what impact ISG15 modification has on a target protein. Unlike ubiquitination, ISGylation does not appear to directly target proteins for proteasome-mediated degradation [29]. Chains of ISG15 do not appear to be formed and no interactions between ISG15 and components of the proteasome have been identified. Based upon the analysis of a limited number of target proteins, ISG15 appears to function by either disrupting the activity of a target protein and/or by altering its localization within the cell [30,31]. The mechanism by which this occurs is largely unknown and will remain an important focus of future investigations. Interestingly, many of the potential target proteins identified include proteins that play an important role in immunity and the host antiviral response, such as STAT1, JAK1, RIG-1, ISG56, PKR, and MxA. The recent observation that Herc5 mediates the ISGylation of actively translated proteins would make many of these IFN induced proteins important targets [24]. It would also provide additional support for ISG15 playing a significant role during viral infection. As discussed below, the importance of ISG15 in the host response to viral infection has been confirmed in (1) studies evaluating the impact of ISG15 overexpression/knockdown on viral growth *in vitro*; (2) studies of mice deficient in members of the ISG15 conjugation cascade; and (3) the evaluation of viral immune evasion strategies targeting ISG15.

## 2. Antiviral Activity of ISG15

The impact of the ISG15 pathway has been investigated both *in vitro* and *in vivo* (Table 1). Studies looking at either the overexpression of ISG15 or knockdown of ISG15 using siRNA have implicated it in the regulation of influenza, vaccinia, vesicular stomatitis virus (VSV), Sendai, Japanese encephalitis virus (JEV), as well as in the release of virus-like particles (VLPs) derived from HIV-1 and avian sarcoma leukosis virus (ASLV) [24,32–39]. There has also been a recent report that ISG15 functions as a proviral molecule, augmenting hepatitis C virus (HCV) replication, although the mechanism is yet to be determined [40]. Studies utilizing siRNA that target ISG15 have also shown either increased viral growth (vaccinia, Sendai, NDV) or an ability to partially rescue type I IFN induced inhibition of growth (influenza A, Ebola VLPs, HIV-1) [32,34,38]. In most cases these effects have been modest, with changes in viral growth ranging between 5–20 fold. The mechanism by which ISG15 is regulating viral growth is unknown for the majority of these pathogens. In most overexpression studies, ISG15 and members of its conjugation cascade have been co-expressed, suggesting that the conjugation of ISG15 to target proteins is required for these antiviral effects. An exception is the studies of Ebola virus VLP release, in which the overexpression of only ISG15 inhibited VLP budding [32,39]. Despite these observations with either overexpression or knockdown strategies, no differences in viral growth of influenza A, herpes simplex virus (HSV-1), Sindbis, VSV or wild type vaccinia virus have been reported in cells derived from either ISG15−/− or UbE1L−/− mice [12,41,42]. Furthermore, it is unknown if these *in vitro* effects will translate into differences *in vivo*. For example, while defects in VSV budding were noted in an ISG15 overexpression study, to date, no phenotype has been observed or reported in mice infected with VSV that lack either ISG15 or UbE1L [12,32,42]. Future studies are needed to confirm these phenotypes *in vivo*.

The strongest evidence for ISG15 functioning as an antiviral molecule has come from the analysis of mice in which ISG15 or members of the conjugation cascade have been deleted. The first genetically deficient mouse to be generated in the pathway was the deconjugating enzyme, Ubp43. These mice died around 4 weeks of age from hydrocephalus [43]. Characterization of these mice found them to have increased levels of ISG15 conjugates, increased IFN signal transduction as noted by prolonged STAT-1 phosphorylation, and increased production of ISGs [44,45]. They were also resistant to both viral and bacterial infections, suggesting that the dysregulation of ISG15 conjugation was leading to this phenotype [45]. This conclusion was challenged when the initial characterization of ISG15−/− mice revealed no defect in their development, intact interferon signaling, and normal resistance to VSV and lymphocytic choriomeningitis virus (LCMV) infection [42]. In addition, the phenotypes observed in the Ubp43−/− mice were not abrogated upon the generation of mice lacking both Ubp43 and ISG15, indicating that the phenotype of the Ubp43−/− mouse was independent of ISG15 [46]. Subsequent studies have determined that Ubp43, in addition to its role as an ISG15 deconjugating enzyme, also functions as a negative regulator of type I IFN receptor signaling [47].

Confirmation that ISG15 functions as an antiviral molecule has come from the careful analysis of ISG15^−/−^ mice. ISG15^−/−^ mice, as compared to wild type mice, were more susceptible to infection with Sindbis virus, influenza A and B virus, HSV-1, and murine gammaherpesvirus 68 [41]. While both free ISG15 and ISG15 conjugates were detected during viral infection, ISGylation was shown to be required for ISG15-dependent resistance to Sindbis and influenza virus *in vivo*. The increased lethality seen in ISG15−/− mice infected with Sindbis virus could be rescued by a double subgenomic Sindbis virus expressing wild type ISG15, but was not rescued by viruses containing mutations in the terminal LRLRGG motif, which abrogated conjugation [41,48,49]. In addition, mice lacking the ISG15 E1 enzyme, UbE1L, which still express free ISG15, but fail to form ISG15 conjugates, were also susceptible to both Sindbis virus and influenza virus infection [48,50]. Studies of vaccinia virus found that ISG15−/− mice displayed no differences in lethality and had a minimal increase in viral loads following infection with wild type virus [33]. The E3 protein was identified as an ISG15 antagonist, which inhibited ISG15 conjugation [33]. Infection of ISG15−/− mice with a mutant virus lacking this ISG15 antagonist did result in increased lethality as compared to WT mice, providing another model in which ISG15 played a role. Therefore in these viral systems the antiviral action of ISG15 is mediated through its conjugation to target proteins. Further investigation is now needed to determine the viral and host proteins that are targeted during infection, and to understand the mechanism by which this modification impacts upon the virus.

## 3. Mechanism(s) of Antiviral Activity

The mechanism by which ISG15 is functioning as an antiviral molecule is unknown, and likely differs between viruses. Recent studies have provided some insight into how ISG15 regulates the host antiviral response to some viruses.

### 3.1. Disruption of Viral Budding

A series of studies looking at viruses that utilize the host ubiquitin machinery for viral egress have implicated ISG15 as an important regulator of this pathway. Both retroviruses and filoviruses utilize the endosomal sorting complex required for transport (ESCRT I–III) pathway for viral egress. Okumura *et al.* were the first to demonstrate that the over-expression of ISG15 mimicked the ability of type I IFN to block the release of HIV particles from cells. Through the utilization of siRNA, they found that this inhibition was due to the activity of ISG15 [38]. ISG15 overexpression inhibited the ubiquitination of both HIV Gag and host Tsg101. This disrupted the interaction between these proteins, which was known to be critical for efficient viral budding [38]. The authors failed to detect ISGylation of either HIV Gag or Tsg101 directly. They proposed that this loss of ubiquitination may lead to a less stable budding complex, and therefore decreased budding.

This observation was followed by two independent studies demonstrating that ISG15 also inhibited Ebola virus VP40 VLP release [32,39]. Budding in this system requires the interaction of the VP40 late domain with members of the host ESCRT pathway (*i.e.* Tsg101). Monoubiquitination of VP40, mediated by the Nedd4 E3 ligase, is thought to be required for efficient release. In studies by both Okumura *et al.* and Malakhova *et al.* the overexpression of ISG15, independent of the conjugation system, inhibited Ebola virus VP40 VLP release. Mechanistically, they found that ISG15 interacted with Nedd4 and abrogated the transfer of ubiquitin from the E2 enzyme to the active site of Nedd4 [32,39]. This prevented Nedd4-mediated ubiquitination of target proteins including Ebola virus VP40. They extended these studies to VSV, a rhabdovirus which contains a similar L-domain and also interacts with Nedd4 ligase for efficient budding [32]. These two studies identified a mechanism by which ISG15 regulated viral budding, and for the first time, implicated unconjugated ISG15 as an antiviral molecule.

An additional mechanism by which ISG15 targets retroviral release from cells late in the budding process has recently been identified. The release of (ASLV) VLPs, similar to HIV-1, was inhibited by the overexpression of ISG15 [37]. The expression of ISG15 inhibited ASLV Gag ubiquitination, supporting the earlier findings. However, the authors were able to reconstitute Nedd4 independent budding by covalently linking either ASLV or HIV-1 Gag proteins to the ESCRT-III protein, CHMP6, allowing ASLV and HIV-1 Gag to enter their respective budding pathways at a late step in the budding process [37]. These constructs were still inhibited by ISG15, suggesting a second mechanism for ISG15 mediated inhibition of viral release. The ectopic expression of ISG15 blocked the association of HIV-1 Gag and ASLV Gag with Vps4, by inhibiting the interaction between Vps4 and its co-activator, LIP5 [37]. This prevented the formation of the oligomeric enzyme needed to disassemble the ESCRT complex and facilitate multiple rounds of budding. The ESCRT-III protein, CHMP5, was noted to be modified by ISG15, and depletion of CHMP5 by siRNA made the budding of ASLV and HIV-1 VLPs resistant to ISG15 inhibition [37].

Together, these studies provide the first insight into one mechanism by which ISG15 may regulate viral infection, by targeting multiple stages of budding for viruses that utilize ubiquitin machinery and ESCRT pathway. In most cases, these studies were performed using overexpression systems. Whether these interaction/modifications also take place, and are significant, during viral infection in cells or in animal models remains to be determined.

### 3.2. Modification of Viral Proteins

Host proteins have clearly been identified as targets of ISGylation, although as discussed above, the impact of this modification is still largely debated. A persistent question had been whether viral proteins are also targeted for ISG15 modification. Recently two groups have demonstrated the modification of the influenza A viral protein, NS1, which functions as an IFN antagonist, during viral infection [51,52]. In the study by Zhao *et al.*, the authors determined that during viral infection the dominant lysine that was modified by ISG15 was at position 41 in the RNA-binding domain of NS1, and that ISGylation at this site disrupted its association with importin-α, a protein required for the nuclear import of NS1 [52]. The generation of a recombinant virus in which this lysine residue was mutated (K41R), resulted in greatly reduced ISGylation of NS1 during viral infection, and demonstrated that the ISGylation of NS1 inhibited replication of influenza in IFN-β-treated cells [52]. In a second study by Tang, *et al.* the authors identified seven lysines (K20/41/108/110/126/217/219) in the PR8 NS1A as potential ISGylation sites, with K126 and K217 found to be critical *in vivo* [51]. The generation of mutant viruses carrying the K → R mutations at either of these lysine residues resulted in augmented viral growth in cultured cells and increased virulence *in vivo* [51]. These studies provided the first evidence that viral proteins are also targeted for ISGylation and that this modification impacts virulence. Future experiments are needed to determine whether NS1 is the only influenza viral protein modified during influenza virus infection. Finally, a recent study by Durfee *et al.* found that the E3 ligase, Herc5, associated with polyribosomes, and therefore targeted newly synthesized proteins for modification by ISG15 [24]. The authors proposed that viral proteins would be a primary target of ISGylation, and they went on to demonstrate that the HPV16 L protein was ISGylated in transfection experiments [24]. When an HPV pseudovirus system was utilized to determine if ISGylation impacted infectivity, the authors found that HPV16 pseudovirus generated in cells cotransfected with ISG15, E1, E2, and E3 resulted in decreased infectivity of the virus [24]. It remains to be seen if modification of the L protein occurs during viral infection. Taken together, these results support viral proteins as targets for ISG15. Additional work is needed to determine if this modification accounts for the entire phenotype seen in mice lacking ISG15, or if host protein modification also contributes to the antiviral action of ISG15.

### 3.3. Modification of Host Proteins

Many of the target proteins identified by proteomic screens include proteins that play an important role in immunity, such as STAT1, JAK1, RIG-1, ISG56, PKR, and MxA [25–27]. Modification of these proteins may contribute to antiviral activity against specific viruses, although at this point in time their contribution to the antiviral state is unclear, due, in part, to the unknown fate of the vast majority of ISGylated target proteins. Recently, the mechanism by which ISG15 alters the function of several substrates has been identified. It appears that ISG15 can directly impact the function of the modified protein or it indirectly impacts its function by disrupting ubiquitination. For example, ISG15 negatively regulates the scaffold protein filamin B [30]. Unmodified filamin B facilitates type I IFN-induced apoptosis by recruiting RAC1, MEKK1, MKK4, and JNK1. ISGylation of filamin B, however, releases RAC1, MEKK1, and MKK4 from the scaffold, preventing IFN-β-dependent JNK activation and apoptosis [30]. It has been suggested that this mechanism may protect uninfected bystander cells from IFN-mediated apoptosis, but this hypothesis awaits further experimentation. In contrast, ISG15 conjugation positively regulates the transcription factor IRF3 that promotes type I IFN induction [36,53]. ISGylation of IRF3 abolishes its binding to Pin1, a protein that promotes IRF3 ubiquitination and subsequent degradation [36]. ISG15-dependent inhibition of the IRF3-Pin1 interaction correlates with decreased IRF3 degradation and increased IFN-β induction [36]. ISG15 modification of several ubiquitin E2s (Ubc13, UbcH6, and UbcH8) has been shown to disrupt the ability of these E2s to form thioester bonds with ubiquitin, and therefore disrupts the ubiquitination of downstream targets [31,54]. The contributions of these and other ISG15 modified host proteins to the antiviral activity of ISG15 are still under investigation.

## 4. Immune Evasion Strategies Targeting ISG15

Host pathways that play a significant role in the antiviral response are often targeted by viruses. Viral immune evasion strategies have been identified that target the ISG15 pathway (Figure 1), providing further support for the importance of this pathway in the host anti-viral response. To date, all of the evasion strategies have targeted ISG15 conjugation by either inhibiting the formation of conjugates or by disrupting the conjugates once formed.

The first identified immune evasion strategy targeting the ISG15 pathway was described by Robert Krug and colleagues while studying interacting partners for the influenza viral protein, NS1 [11]. They determined that while the infection of cells at a high MOI with various influenza B viral strains upregulated free ISG15, no ISG15 conjugates were detected. The mechanism for this inhibition was mediated by the NS1/B gene, which non-covalently bound to ISG15 and inhibited the ability of the ISG15 E1, UbE1L, from interacting with and subsequently activating ISG15, thereby inhibiting conjugate formation [11]. While the NS1 proteins of both influenza A and B viruses are antagonists of IFN, only B/NS1, and not A/NS1 retained the ability to bind to ISG15 and inhibit conjugate formation [11]. Despite this evasion strategy, the ISG15−/− and UbE1L−/− mice are highly susceptible to influenza B virus infection [41,50]. This appears to be due largely to the species specificity of the B/NS1-ISG15 interaction, with B/NS1 binding only to human and non-human primate ISG15, but not to murine or canine ISG15 [20,55]. This may provide important insight into the more restricted host range of influenza B viruses as compared to influenza A viruses.

In addition to influenza B virus, vaccinia virus has also been shown to encode a viral protein that antagonizes ISG15. In this case the E3 protein, which is known to antagonize the host IFN response by several mechanisms (inhibiting PKR, RNaseL and blocking IRF3 activation), was also shown to bind to ISG15 through its C-terminal domain [33]. Once again this protein appears to inhibit ISG15 conjugate formation, although the precise mechanism remains to be determined. In this model, ISG15−/− mice display almost no differences from WT mice following infection with wild type vaccinia virus. However, deletion of the E3 protein from the virus resulted in increased virulence in the ISG15−/− mice, demonstrating its role in the host response, and the efficacy of the immune evasion strategy mounted by vaccinia virus [33].

In contrast to the first two examples, the remaining strategies have taken advantage of the protease activity of different viruses to hydrolyze both ubiquitin and ISG15 from target proteins. The first strategy identified is employed by several viruses with ovarian tumor domain (OTU)-containing proteases [56]. The OTU domain family has recently been identified as deubiquitinating (DUB) enzymes comprised of a group of cysteine proteases which participate in substrate-specific deubiquitination. The L proteins from both Crimean Congo hemorrhagic fever virus (CCHFV) and Dugbe virus (DUGV), as well as the nsP2 proteins from the arteriviruses, equine arteritis virus (EAV) and porcine respiratory and reproductive syndrome virus (PRRSV), contain OTU domains. Unlike their mammalian counterparts (Cezanne and A20) which only had deubiquitinating activity against specific substrates, these viral proteins were found to globally deconjugate both ubiqitin and ISG15 chains, via their cysteine protease activity, but had no activity against SUMO-2 or SUMO-3 conjugates [56]. Expression of these viral OTU domains through either the generation of a transgenic mouse expressing the CCHFV L protein or by overexpressing this protein directly from a recombinant Sindbis virus was able to inhibit the antiviral activity of ISG15 [56]. In addition, these viral OTU proteases were able to inhibit NF-κB dependent signaling [56]. Finally, in addition to OTU domain containing proteases, recent work looking at the papain-like proteases of both the SARS coronavirus (SARS-CoV PLpro) and the human coronavirus NL63 (HCoV-NL63 PLP2) proteins has found that both can deubiquitinate and deISGylate target proteins [57,58]. The significance of this activity during viral infection is still under investigation. In both cases, this viral immune evasion strategy allows these viruses to evade both the IFN and TNFα signaling pathways, both of which play a critical role in the host response to viral infection.

## 5. Conclusion

Significant progress has been made in identifying the components of the ISG15 conjugation cascade, understanding how these proteins are induced during viral infection, and identifying potential targets for ISGylation. While ISG15 may regulate a variety of cellular processes, it appears to play a critical role in the host response to viral infection. This is supported by studies in ISG15−/− and UbE1L−/− mice, and also in the identification of viral immune evasion strategies that target the ISG15 pathway. Several viral systems appear to be regulated by ISG15, based upon both *in vitro* and *in vivo* studies. Recent efforts have begun to dissect the mechanism by which ISG15 is regulating these responses. However, despite this progress many questions still remain and will be the focus of future investigations. First, for many of the viral systems, observations made from either overexpression studies or knockdown studies will need to be confirmed *in vivo*. Second, additional mechanistic studies will be necessary to understand how ISG15 is regulating such a diverse group of viruses. Only after this information is in hand will we be able to develop therapeutics that target this pathway. Third, while viral proteins can be ISGylated, at least during influenza virus infection, it remains unclear if the antiviral effects of ISG15 are due solely to modification of viral proteins, or if the modification of host proteins also contributes to this antiviral effect. Fourth, the report by Durfee *et al.* suggests that only actively translated proteins will be targeted for ISGylation, at least by Herc5 [24]. These would include the many genes that are induced by type I IFN, and will be expressed in cells which are not virally infected. The impact of ISGylation on these proteins and what is their fate remains unanswered. Fifth, to date the antiviral activities of ISG15 and the identified viral immune evasion strategies have targeted ISG15 conjugation. However, studies with the Ebola VLPs suggest that free, unconjugated ISG15 may mediate its antiviral effects [32,39]. Unanchored ubiquitin chains have recently been shown to modify both the NF-kB and RIG-I signaling pathways [59,60]. Therefore, evaluating the potential activity of unconjugated ISG15, both within the cell, and that which is released from the cell, will be of interest. Finally, IFNs have pleotropic effects on the immune response. Determining whether ISG15 plays a role in these responses could have important consequences for the development of future therapeutic and vaccine strategies.

## Figures and Tables

**Table 1:** Studies evaluating the antiviral activity of ISG15.

**Virus**	**Method**	**Cell Type**	**Viral Growth**	**Reference**
Influenza A (A/WNS/33)	ISG15−/−	MEFs	No difference	[41]
Influenza A (A/Udorn/72)	siRNA (ISG15 + UbE1L)	Calu3	Rescued IFN induced inhibition of growth by 10–20 fold at 4 hrs.	[34]
Influenza A (A/PR8/34)	siRNA (ISG15) or siRNA (Herc5)	A549	4 fold increase in viral infectivity	[51]
Vaccinia WR	ISG15−/−	MEFs	No difference	[33]
	ISG15 overexpression	ISG15−/− MEFs	5 fold decrease in viral titers	[33]
	siRNA (ISG15)	WT MEFs	15 fold increase in viral titers	[33]
Vaccinia virus (ΔE3L)	ISG15−/−	ISG15−/− MEFs	25 fold increase in viral titers	[33]
	ISG15 overexpression	ISG15−/− MEFs	25 fold decrease in viral titers	[33]
	siRNA (ISG15)	WT MEFs	15 fold increase in viral titers	[33]
VSV (Indiana)	ISG15−/−	ISG15−/− MEFs	No difference	[42]
VSV	UbE1L−/−	UbE1L−/− MEFs	No difference in VSV protection assay	[12]
VSV-(Indiana)	ISG15 overexpression	293 T cells	10 fold decrease in viral titers	[32]
VSV-PY>A4 mutant	ISG15 overexpression	293 T cells	No effect	[32]
Ebola VP40 VLPs	ISG15 overexpression	293 T cells	Inhibited Ebola VP40 VLP release	[32]
	siRNA (ISG15)	293 T cells	Rescued IFN induced inhibition of Ebola VP40 VLP budding	[32]
Ebola VP40 VLPs (Zaire strain)	ISG15 overexpression	293 T cells	Inhibited Ebola VP40 VLP release	[39]
	ISG15−/−	ISG15−/− MLFs	2 fold increase in VP40 release from cells expressing HA-VP40	[39]
HIV-1 provirus (NL43)	ISG15 overexpression	293 T cells	Inhibited release of HIV	[38]
	siRNA (ISG15)	293T cells	Rescued IFN mediated inhibition of HIV release	[38]
ASLV Gag VLP	ISG15 overexpression	293/E cells	Inhibited ASLV VLP release	[37]
HIV Gag VLP	ISG15 overexpression	293/E cells	Inhibited HIV VLP release	[37]
SeV	siRNA (ISG15 or Herc5)	HEK293	5 fold increase in viral titers	[36]
NDV-GFP	siRNA (Herc5)	HEK293	10 fold increase in GFP+ cells	[36]
JEV (strain T1P1)	ISG15 overexpression	Te-671	10–50 fold decrease in viral titers	[35]
HPV pseudovirus	ISG15 overexpression	293 T cells	Decreased infectivity of pseudovirus generated in ISG15 expressing cells	[24]
HCV replicon (genotype 1b replicon I_377_/NS3-3)	siRNA (ISG15)	MH1 cells or con1 cells	Decreased HCV replication as assessed by RT-PCR.	[40]
HCV (J6/JFH-1)	siRNA (ISG15)	Huh 7.5 cells	Decreased HCV replication	[40]
	ISG15 overexpression	Huh 7.5 cells	Increase in replication	[40]
Influenza A virus (WSN/33/A)	ISG15−/− mice	Increased	N.T.	[41]
Influenza B virus (B/Lee/40)	ISG15−/−mice	Increased	Increased 3–4 logs	[41]
	UbE1L−/− mice	Increased	Increased 3–4 logs	[50]
Influenza B virus (B/Yamagata/88)	ISG15−/−mice	Increased	Increased 3–4 logs	[50]
	UbE1L−/− mice	Increased	Increased 3–4 logs	[50]
Influenza B virus (B/Yamagata/73)	ISG15−/−mice	N.T.	Increased 2–3 logs	[50]
	UbE1L−/− mice	N.T.	Increased 2–3 logs	[50]
Sindbis virus (dsTE12Q)	ISG15−/−mice	Increased	N.T.	[41,48]
dsTE12Q-ISG15 LRLRGG	ISG15−/− mice	Protected from lethality		[48]
dsTE12Q-ISG15 LRLRAA	ISG15−/− mice	No protection		[48]
dsTE12Q-ISG15 LALRGG	ISG15−/− mice	No protection		[49]
Sindbis virus (dsTE12Q)	UbE1L−/−mice	Increased	N.T.	[49]
HSV-1 (strain 17)	ISG15−/− mice	Increased	N.T.	[41]
γHV68	ISG15−/− mice	No difference	Increased 10 fold	[41]
Vaccinia virus (WR)	ISG15−/− mice	No difference	Increased 3 fold	[33]
Vaccinia virus (VVΔE3L)	ISG15−/− mice	Increased	None detected	[33]
Vaccinia virus (VVE3LΔ26C)	ISG15−/− mice	Increased	No difference	[33]
VSV (Indiana strain)	ISG15−/− mice	No difference	N.T.	[42]
LCMV (WE strain)	ISG15−/− mice		No difference	[42]
LCMV (Armstrong strain)	UbE1L−/− mice	No difference	N.T.	[12]
HBV (pSP65-ayw1.3 genome)	UbE1L−/− mice Injection of naked plasmid DNA	N.T.	No difference in viral replication	[61]

Vesicular stomatitis virus (VSV), Human immunodeficiency virus-1 (HIV-1), Sendai virus (SeV), Newcastle disease virus- green fluorescent protein (NDV-GFP), Avian sarocoma leukosis virus (ASLV), Japanese encephalitis virus (JEV), human papillomavirus (HPV), hepatitis C virus (HCV), gammaherpes virus68 (γHV68), herpes simplex virus-1 (HSV-1), lymphocytic choriomeningitis virus (LCMV) hepatitis B virus (HBV). Lethality and viral titers were compared to responses in WT mice; N.T. = not tested/reported.
